# Head-to-head comparison of the efficacy of Xpert MTB/RIF Ultra and Xpert MTB/RIF for the diagnosis of tuberculous pleurisy: A systematic review and meta-analysis

**DOI:** 10.1097/MD.0000000000029363

**Published:** 2022-05-27

**Authors:** Wenfeng Yu, Yanqin Shen, Pengfei Zhu, Da Chen

**Affiliations:** a Zhejiang Tuberculosis Diagnosis and Treatment Center, Affiliated Hangzhou Chest Hospital, Zhejiang University School of Medicine, Zhejiang Chinese Medicine and Western Medicine Integrated Hospital, Hangzhou, Zhejiang, China; b Department of Thoracic Surgery, Cancer Hospital of the University of Chinese Academy of Sciences, Hangzhou, Zhejiang, China.

**Keywords:** diagnostic accuracy, meta-analysis, tuberculous pleurisy, Xpert MTB/RIF, Xpert MTB/RIF Ultra

## Abstract

**Background:**

The aim of this study was to evaluate the diagnostic accuracy of Xpert MTB/RIF Ultra (Xpert Ultra) and Xpert MTB/RIF (Xpert) for the diagnosis of tuberculous pleurisy (TBP) head-to-head using meta-analysis method.

**Methods:**

On May 12, 2021, we searched multiple databases for reports that used Xpert Ultra and Xpert for TBP diagnosis head-to-head and screened eligible studies for inclusion. Accuracy of Xpert Ultra and Xpert were compared to that of the composite reference standard (CRS) and culture. When heterogeneity was evident, sources of heterogeneity were explored using subgroup analyses, sensitivity analysis, and meta-regression analyses.

**Results:**

Five articles met the inclusion criteria for meta-analysis. When results from different specimens or different reference standards were reported in the same article, we analyzed them as separate studies. Thus, 6 studies compared Xpert Ultra and Xpert with CRS, 5 studies compared Xpert Ultra and Xpert with culture. Pooled sensitivity and specificity of Xpert Ultra were 52% and 98% compared to CRS, and 82% and 77% compared to culture. Pooled sensitivity and specificity of Xpert were 22% and 99% compared to CRS, and 48% and 94% compared to culture. Significant heterogeneity in sensitivity was observed compared to CRS.

**Conclusion:**

The sensitivity of Xpert Ultra was moderate but better than that of the Xpert; however, its specificity was lower. The role of Xpert Ultra and Xpert in the early and rapid diagnosis of TBP was limited.

## 1 Introduction

Tuberculosis (TB) remains a serious public health problem worldwide with 10.4 million new cases and 1.4 million deaths recorded in 2018.^[1]^ Furthermore, death due to TB is among the top 10 causes of death and mortality is still high in developing countries, especially among TB patients coinfected with human immunodeficiency virus.^[2]^*Mycobacterium* tuberculosis (MTB) is the etiological agent and most often invades the lungs to cause lung disease, which is called pulmonary tuberculosis (PTB).^[3]^ In contrast, TB outside the lung tissue is called extrapulmonary tuberculosis (EPTB) and accounts for approximately 15% of all reported cases.^[1,4]^ The 2 most common forms of EPTB are lymph node TB and tuberculous pleurisy (TBP).^[4]^ TBP is most commonly diagnosed based on analysis of pleural effusion, but as it needs to be obtained invasively via a thoracostomy and is characterized by paucibacillary, associated tests such as *Mycobacterium* culture and microbiological examination have limited sensitivity and specificity.^[5,6]^ Thus, early diagnosis of TBP remains a challenge for clinicians.

Currently, the Xpert MTB/RIF (Xpert) is the most commonly used molecular test for TB worldwide.^[7]^ This method has good sensitivity and specificity for the early diagnosis of PTB and was approved by the World Health Organization for PTB diagnosis in 2010.^[8]^ Importantly, as follow-up studies demonstrated that this method could also effectively detect EPTB (such as tuberculous meningitis and lymph node TB ), it was approved by the World Health Organization in 2013 for diagnosis of EPTB.^[9]^ However, multiple studies have reported unsatisfactory diagnostic accuracy for the Xpert in paucibacillary specimens such as pleural effusions.^[10]^ To address these issues, Cepheid developed the next-generation Xpert MTB/RIF Ultra (Xpert Ultra), which shares a diagnostic platform with the existing Xpert system.^[11]^ The Ultra assay incorporates IS1081 and IS6110 as additional MTB target sequences, resulting in a detection limit that is significantly lower than that of the Xpert, thereby improving assay performance.^[12,13]^ Notably, several studies have reported improved diagnostic potential of the Xpert Ultra in paucibacillary specimens.^[14–16]^ However, comparison of the diagnostic efficacy of Xpert Ultra and Xpert for TBP is still uncertain,^[17,18]^ we performed a systematic review and meta-analysis to synthesize evidence on the diagnostic ability of Xpert Ultra in TBP, and head-to-head comparison of the diagnostic accuracy of Xpert Ultra and Xpert systems in the same population.

## 2 Methods

### 2.1 Design and registration

This systematic review and meta-analysis was based on the stated purpose of the study and followed Preferred Reporting Items for Systematic Reviews and Meta Analyses (PRISMA) guidelines,^[19]^ issued by the EQUATOR network. We had registered the protocol on the International Platform of Registered systematic Review and Meta-Analysis Protocols (INPLASY; registration number: INPLASY202080047).^[20]^ Furthermore, ethical approval was waived for systematic review and meta-analysis.

### 2.2 Information sources

On May 12, 2021, we searched the Cochrane Library, Embase, PubMed, China National Knowledge Infrastructure (CNKI), and the Wanfang database for studies that had used the Xpert Ultra and Xpert for TBP diagnosis. We also scrutinized references cited in reviews for potential studies.

### 2.3 Search strategy

The search strategy was developed by Wenfeng Yu and Yanqin Shen. There were no language or temporal restrictions in the search strategy. The search strategy used in PubMed is as follows:

#1 “Tuberculosis, Pleural”[Mesh] OR “Pleural Tuberculoses” OR “Pleural Tuberculosis” OR “Pleural TB” OR “Tuberculoses, Pleural” OR “Pleurisy, Tuberculous” OR “Pleurisies, Tuberculous” OR “Tuberculous Pleurisies” OR “Tuberculous Pleurisy” OR “Pleural Effusion”[Mesh] OR “Effusion, Pleural” OR “Effusions, Pleural” OR “Pleural Effusions” OR “Extrapulmonary tuberculosis” OR “Extra pulmonary tuberculosis”
#2 “Xpert Ultra” OR “GeneXpert Ultra”
#3 “Xpert” OR “GeneXpert”
#4 #1 AND #2 AND #3

Similar search strategies were used in Embase, Cochrane Library, Wanfang databases, and CNKI.

### 2.4 Eligibility criteria

**Type of study:** Any study that had evaluated the accuracy of Xpert Ultra and Xpert for TBP.

**Participants:** Participants diagnosed with TBP using Xpert Ultra without any limitations on gender, age, or nationality.

**Index test:** Xpert Ultra was considered as index test.

**Comparator test:** Xpert was considered as comparator test.

**Outcomes:** Sensitivity and the specificity of the Xpert Ultra and Xpert were the main outcomes.

**Target conditions:** Full text original studies that evaluated the use of Xpert Ultra and Xpert for TBP diagnosis with appropriate and clear reference standards and sufficient data to directly extract or calculate true positive (TP), false positive (FP), false negative (FN), and true negative (TN) values were included. Case reports, articles written in languages other than Chinese and English, studies with <10 specimens, conference reports, and abstracts without full text were excluded.

**Reference standards:** Culture or a composite reference standard (CRS) was used as the reference standard. The CRS comprised clinical symptoms, imaging features, biochemical analysis of pleural effusion, MTB smears, culture, pleural biopsy histopathology, and response to anti-TB therapy. TBP was defined as positivity for some or all of these factors, while negative for all of these factors was considered non-TBP.

### 2.5 Literature screening and selection

Potential studies were imported into the Endnote X9.2 (Clarivate) literature management software. Two investigators (Wenfeng Yu and Yanqin Shen) independently reviewed titles and abstracts, and then the full text to evaluate suitability for inclusion. Any disputes between the 2 investigators were resolved by negotiation with a third investigator (Da Chen).

### 2.6 Data extraction

Data extracted included name of first author; year of publication; country in which the study had been conducted; TP, FP, FN, and TN values for Xpert Ultra; reference standard; patient selection method; specimen type and processing steps (e.g., homogenization); and specimen condition. If an article also compared the diagnostic accuracy of Xpert Ultra and Xpert systems using different types of sample, then relevant data for different types of sample were similarly extracted. All necessary data from the included articles were independently extracted by the same 2 investigators who screened and selected articles. The extracted data were cross-checked for accuracy and any discrepancies were resolved by discussion with a third investigator (Da Chen). Data that compared divergent reference standards were treated as separate studies and used as such for analyses.

### 2.7 Quality evaluation

Two investigators used a revised version of the Quality Assessment of Diagnostic Accuracy Studies (QUADAS-2) tool to independently assess study quality of included studies and to compare it with that of the 2 reference standards (CRS and culture).^[21]^ Any disputes between the 2 investigators were resolved by negotiation with a third member of the research team (Da Chen). The Preferred Reporting Items for Systematic Reviews and Meta-Analysis for Diagnostic Test Accuracy (PRISMA-DTA) guideline do not mandate a systematic review and meta-analysis of diagnostic test accuracy studies to assess publication bias,^[22]^ therefore, we did not assess publication bias.

### 2.8 Data synthesis and statistical analysis

Values for Xpert Ultra and Xpert parameters such as TP, FP, FN, and TN were extracted from each included study and were used to calculate estimated pooled sensitivity and specificity for Xpert Ultra and Xpert systems, along with 95% confidence intervals (CIs). Forest plots for sensitivity and specificity were generated for each study. *I*^2^ statistics were calculated to assess heterogeneity between the studies, wherein 0% indicated no observed heterogeneity while values greater than 50% implied substantial heterogeneity.^[23]^ Subgroup analyses, sensitivity analysis, and meta-regression analyses were used to identify potential sources of heterogeneity.^[24]^ RevMan version 5.3 (Cochrane Collaboration, Oxford, United Kingdom) was used to generate forest plots for sensitivity and specificity with 95% CI for all included studies. STATA (version 15.0; Stata Corp., College Station, TX) with the *midas* command package was used to carry out meta-analyses and meta-regression analyses.

## 3 Results

### 3.1 Identification of studies and study characteristics

**Figure 1:**
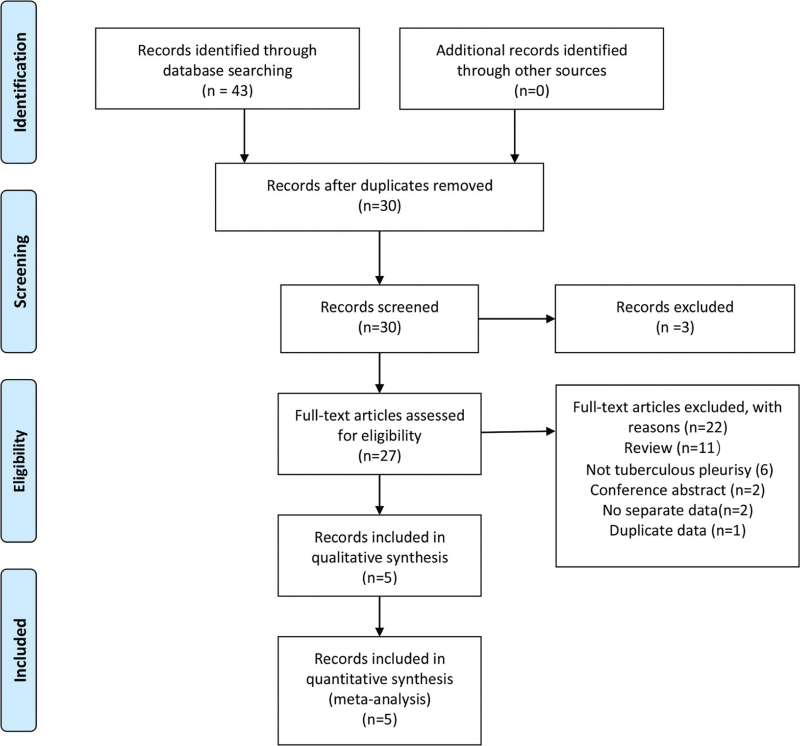
Literature retrieval flow chart. A total of 4, 8, 12, 2, and 17 articles were identified in the Cochrane Library, Embase, PubMed, the Wanfang databases, and China National Knowledge Infrastructure (CNKI), respectively.

Our search strategy identified 43 candidate articles and subsequent screening led to the identification of 5 articles that met the inclusion criteria for further analysis.^[17,18,25–27]^ The literature search flowchart is presented in Figure 1. Kappa values for consistency between the 2 investigators during literature screening and data extraction was 0.659 (95% CI, 0.349–0.969). Among the 5 studies, all articles reported research conducted in high TB incidence countries. All articles were published in English. We excluded 1 article due to data duplication^[28]^ and 2 articles were excluded as they did not report specific data on TBP.^[29,30]^ Among the included articles, minimum and maximum sample sizes were 61 and 292, respectively. Specimen types used for diagnosis included pleural fluid or pleural tissue; 1 study used pleural fluid and biopsy specimens,^[17]^ 1 study used pleural tissue, the others used pleural fluid specimens.

When results from different types of sample or different reference standards were reported in the same article, we analyzed them as separate studies. Thus, we used data from 6 studies compared Xpert Ultra and Xpert with CRS, and 5 studies compared Xpert Ultra and Xpert with culture (Table 1). Total sample size for studies that compared CRS with Xpert Ultra and Xpert was 799 patients, while it was 667 for those that compared culture with Xpert Ultra and Xpert.

**Table 1:** Characteristics of the included studies.

Test	Author	Year	County	Sample type	Reference	Research type	Decontaminate method	Sample condition	Homogenisation	SR	Patient selection method
Xpert Ultra	Meldau, R.a	2019	South Africa	Pleural fluid and biopsy	CRS	Prospective	No	Frozen	Mechanical	2:1	Convenience
	Guirong Wang.a	2019	China	Pleural fluid	CRS	Prospective	NALC-NaOH	Fresh	Mechanical	2:1	Consecutive
	Xiaocui Wu.a	2019	China	Pleural fluid	CRS	–	No	Fresh	No	4:1	Convenience
	Guirong Wang.a	2020	China	Pleural fluid	CRS	Prospective	NALC-NaOH	Frozen	Mechanical	2:1	Consecutive
	Shan Gao.a	2021	China	Pleural fluid	CRS	Prospective	No	Frozen	Mechanical	2:1	Consecutive
	Shan Gao.b	2021	China	Pleural tissue	CRS	Prospective	No	Frozen	Mechanical	2:1	Consecutive
	Guirong Wang.b	2019	China	Pleural fluid	Culture	Prospective	NALC-NaOH	Fresh	Mechanical	2:1	Consecutive
	Xiaocui Wu.b	2019	China	Pleural fluid	Culture	–	No	Fresh	No	4:1	Convenience
	Guirong Wang.b	2020	China	Pleural fluid	Culture	Prospective	NALC-NaOH	Frozen	Mechanical	2:1	Consecutive
	Shan Gao.c	2021	China	Pleural fluid	Culture	Prospective	No	Frozen	Mechanical	2:1	Consecutive
	Shan Gao.d	2021	China	Pleural tissue	Culture	Prospective	No	Frozen	Mechanical	2:1	Consecutive
Xpert	Meldau, R.b	2019	South Africa	Pleural fluid and biopsy	CRS	Prospective	No	Frozen	Mechanical	2:1	Convenience
	Guirong Wang.c	2019	China	Pleural fluid	CRS	Prospective	NALC-NaOH	Fresh	Mechanical	2:1	Consecutive
	Xiaocui Wu.c	2019	China	Pleural fluid	CRS	–	No	Fresh	No	4:1	Convenience
	Guirong Wang.c	2020	China	Pleural fluid	CRS	Prospective	NALC-NaOH	Frozen	Mechanical	2:1	Consecutive
	Shan Gao.e	2021	China	Pleural fluid	CRS	Prospective	No	Frozen	Mechanical	2:1	Consecutive
	Shan Gao.f	2021	China	Pleural tissue	CRS	Prospective	No	Frozen	Mechanical	2:1	Consecutive
	Guirong Wang.d	2019	China	Pleural fluid	Culture	Prospective	NALC-NaOH	Fresh	Mechanical	2:1	Consecutive
	Xiaocui Wu.d	2019	China	Pleural fluid	Culture	–	No	Fresh	No	4:1	Convenience
	Guirong Wang.d	2020	China	Pleural fluid	Culture	Prospective	NALC-NaOH	Frozen	Mechanical	2:1	Consecutive
	Shan Gao.g	2021	China	Pleural fluid	Culture	Prospective	No	Frozen	Mechanical	2:1	Consecutive
	Shan Gao.h	2021	China	Pleural tissue	Culture	Prospective	No	Frozen	Mechanical	2:1	Consecutive

CRS = composite reference standard, NALC-NaOH = N-acetyl-L-cysteine-sodium hydroxide, SR = sample ratio, Xpert = Xpert MTB/RIF, Xpert Ultra = Xpert MTB/RIF Ultra.

### 3.2 Study quality

**Figure 2:**
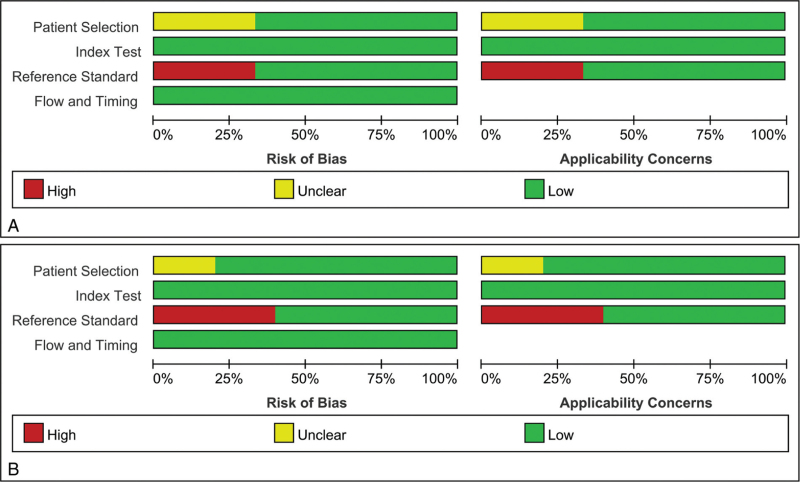
Methodological quality graphs (risk of bias and applicability concerns) as percentages across the included studies of the Xpert Ultra and Xpert. (A) compared with composite reference standard; (B) compared with culture. Xpert = Xpert MTB/RIF, Xpert Ultra = Xpert MTB/RIF Ultra.

Results of quality assessment of the included studies were provided in Figure 2; comparisons were between CRS or culture and Xpert Ultra and Xpert, respectively. Major sources of bias included methods of patient selection and the reference standard used. The flow and timing of the risk of bias from the index test was judged to be relatively low.

### 3.3 Diagnostic accuracy of Xpert Ultra and Xpert for TBP

**Figure 3:**
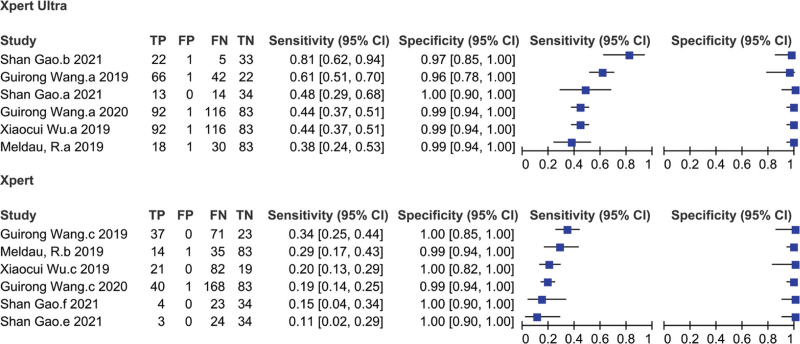
Forest plot of Xpert MTB/RIF Ultra and Xpert MTB/RIF sensitivity and specificity for tuberculous pleurisy compared with a composite reference standard.

Diagnostic accuracy of Xpert Ultra was evaluated based on data from 6 studies, which corresponded to 799 patients. Compared to CRS, minimum and maximum sensitivity values of Xpert Ultra for TBP diagnosis were 38% (24%–53%) and 81% (62%–94%), respectively, and pooled sensitivity was 52% (41%–63%) with *I*^2^ = 80%. Minimum and maximum specificity of Xpert Ultra were 89% (67%–99%) and 100% (90%–100%), respectively, and pooled specificity was 98% (95%–99%) with *I*^2^ = 50% (Fig. 3). Significant heterogeneity in sensitivity was observed, and the heterogeneity of specificity was not obvious.

Diagnostic accuracy of Xpert for TBP was reported in the same 6 studies, which included data from 799 patients. Compared to CRS, minimum and maximum sensitivity of Xpert were 11% (2%–29%) and 34% (25%–44%), respectively, and pooled sensitivity was 22% (17%–29%) with *I*^2^ = 65%. Minimum and maximum specificity of Xpert for TBP were 99% (94%–100%) and 100% (90%–100%), respectively, while pooled specificity was 99% (97%–100%) with *I*^2^ = 0% (Fig. 3). There was significant heterogeneity in sensitivity but insignificant heterogeneity in specificity. Thus, compared to Xpert, Xpert Ultra had greater sensitivity but lower specificity using CRS as gold standard.

**Figure 4:**
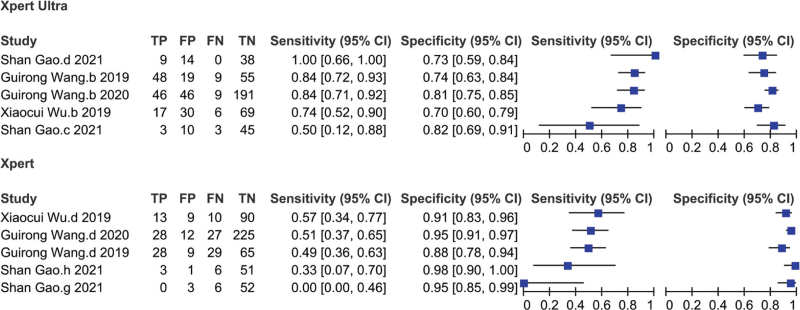
Forest plot of Xpert MTB/RIF Ultra and Xpert MTB/RIF sensitivity and specificity for tuberculous pleurisy compared with culture.

Next, we evaluated the diagnostic accuracy of Xpert Ultra and Xpert compared to culture. Relevant data for Xpert Ultra were reported in 5 studies, which corresponded to 667 patients. Minimum and maximum sensitivity of Xpert Ultra for diagnosing TBP were 50% (12%–88%) and 100% (66%–100%), respectively, and pooled sensitivity was 82% (75%–87%) with *I*^2^ = 46%. Minimum and maximum specificity were 70% (60%–79%) and 82% (69%–91%), respectively, and pooled specificity was 77% (72%–81%) with *I*^2^ = 35% (Fig. 4). There was insignificant heterogeneity in sensitivity and specificity.

The same 5 studies, corresponding to 667 patients, compared diagnostic accuracy of Xpert with that of culture. The sample size in these studies ranged from 61 to 292. Minimum sensitivity and specificity for Xpert were 0% (0%–46%) and 88% (78%–94%), respectively, while maximum sensitivity and specificity were 57% (34%–77%) and 98% (90%–100%), respectively (Fig. 4). The pooled sensitivity and specificity for Xpert compared with culture were 48% (39%–56%; *I*^2^ = 44%) and 94% (90%–96%; *I*^2^ = 47%), respectively. There was insignificant heterogeneity in sensitivity and specificity. Nonetheless, sensitivity of Xpert Ultra was better than that of Xpert, but specificity was lower using culture as gold standard.

Only 2 studies have evaluated the accuracy of Xpert Ultra in rifampicin resistance testing. This corresponded to 55 patients, of whom 11 were rifampicin-resistant and 44 were rifampicin-sensitive. The sensitivity and specificity of Xpert Ultra and Xpert for rifampicin resistance testing were 100%.

As preliminary analysis revealed significant heterogeneity in sensitivity between studies that compared Xpert Ultra and Xpert with CRS, we further analyzed heterogeneity using subgroup analyses, sensitivity analysis, and meta-regression analyses. Subgroup and meta-regression analysis were performed using predefined parameters, namely, patient selection methods, sample type, sample condition, decontamination methods, and homogenization methods used. However, the small number of studies precluded meta-regression analysis (less than10 studies for each study-level variable).

Subgroup analysis showed that the sensitivity and specificity of Xpert Ultra compared with CRS when using pleural effusion specimens, frozen specimens, consecutive specimens, and homogenized specimens by mechanical methods were 49% (41%–57%; *I*^2^ = 68%), 97% (92%–99%; *I*^2^ = 59%); 52% (35%–69%; *I*^2^ = 83%), 99% (96%–100%; *I*^2^ = 0%); 54% (40%–67%; *I*^2^ = 83%), 98% (95%–99%; *I*^2^ = 0%); and 58% (43%–71%; *I*^2^ = 87%), 98% (94%–99%; *I*^2^ = 0%), respectively. The sensitivity and specificity of Xpert with pleural effusion specimens, frozen specimens, consecutive specimens, and homogenized specimens by mechanical means compared with CRS were 22% (16%–30%; *I*^2^ = 76%), 99% (94%–100%; *I*^2^ = 0%); 20% (16%–24%; *I*^2^ = 67%), 99% (97%–100%; *I*^2^ = 0%); 21% (413%–32%; *I*^2^ = 78%), 99% (96%–100%; *I*^2^ = 0%); and 23% (16%–31%; *I*^2^ = 70%), 99% (97%–100%; *I*^2^ = 0%), respectively. These parameters did not reduce the heterogeneity in sensitivity between studies. However, for other parameters, because of the small number of studies included, further analysis for any given situation could not be undertaken.

Sensitivity analysis did not reveal studies with significant heterogeneity, and the study analysis was robustly.

## 4 Discussion

TBP is the second most prevalent form of EPTB and often presents as a pleural effusion that requires thoracentesis for specimen retrieval and subsequent analysis and diagnosis.^[4]^ Pleural effusion is naturally paucibacillary, which reduces the sensitivity of traditional microbiological testing toward TBP diagnosis.^[31]^ Additionally, early diagnosis of TBP is difficult and there are no credible same-day diagnostic tools for TBP.^[17]^ Importantly, treatment delays can worsen prognosis in TBP and lead to complications, such as pyothorax or thoracic narrowing.^[32]^ Thus, there is great demand for accurate and rapid diagnostic tests for TBP that can help improve patient satisfaction and prognosis.

Advances in molecular biomedicine have led to the development of nucleic acid amplification tests (NAATs) for the detection of pathogenic microorganisms, including for TB .^[33]^ By amplifying nucleic acids, NAATs improve nucleic acid detectability and have come to play increasingly important roles in early and rapid diagnosis of pathogenic microorganisms.^[34]^

Xpert, an excellent example of NAATs, greatly improves diagnostic accuracy of PTB and some types of EPTB, thereby enabling an early diagnosis of TB. Xpert is an automated half-nest real time-polymerase chain reaction that yields results in less than 2 hours.^[35]^ Although Xpert performs well in most cases of TB, it does not do so in paucibacterial conditions such as PTB.^[10]^ Further, even though the Xpert has a high diagnostic specificity for PTB, its sensitivity remains unsatisfactory, and sensitivity is relatively more important in TB diagnosis.

The second generation of Xpert, i.e., Xpert Ultra, was created to improve detection efficiency of paucibacillary TB samples. Xpert Ultra is an improved version of Xpert that can still be used on Xpert platform. Specifically, Xpert Ultra is a full-nest real time-polymerase chain reaction with 2 multicopy gene insertions as MTB target sequences, namely, IS1081 and IS6110, along with a higher-capacity reaction cassette. These improvements have significantly lowered its limit of detection; the limit of detection of Xpert Ultra for MTB H37Rv in sputum has reduced to 15.6 colony forming unit/mL from 112.6 colony forming unit/ml for Xpert.^[12]^ Simultaneously, Xpert Ultra uses melt curve analysis to detect rifampicin resistance. Furthermore, this method can provide negative results in 65 minutes and positive results in 77 minutes, which is faster than Xpert.^[11]^ The characteristics of the 2 tests were shown in Table 2. Current evidence also indicates that Xpert Ultra may be promising for use in paucibacillary TB such as smear-negative PTB and EPTB.^[15,36]^ The use of Xpert Ultra had also been evaluated in TBP, but its diagnostic accuracy varies among these studies and there has been no independently systematic evaluation of these studies till date. Thus, this systematic review and meta-analysis aimed to head-to-head comparison of the efficacy of Xpert Ultra and Xpert for the diagnosis of TBP.

**Table 2:** Characteristics of the 2 tests and the sensitivity and specificity of this study.

Test	NAAT type	Target sequences	LOD	Reaction cartridge capacity	Detect rifampicin resistance	Time to report results	Pooled sensitivity compared to culture	Pooled specificity compared to culture	Pooled sensitivity compared to CRS	Pooled specificity compared to CRS
Xpert	Half-nest RT-PCR	rop B	112.6 CFU/mL	Low	Yes	2 h	48%	94%	22%	99%
Xpert Ultra	Full-nest RT-PCR	rop B, IS 1081, IS6110	15.6 CFU/mL	High	Yes	65–77 min	82%	77%	52%	98%

CFU = colony forming unit, CRS = composite reference standard, LOD = limit of detection, NAAT = nucleic acid amplification test, RT-PCR = real time-polymerase chain reaction, Xpert = Xpert MTB/RIF, Xpert Ultra = Xpert MTB/RIF Ultra.

We included 6 studies that compared Xpert Ultra and Xpert with CRS, and 5 studies that compared Xpert Ultra and Xpert with culture. The pooled sensitivity and specificity of Xpert Ultra for TBP compared with CRS and culture were 52%, 98%, and 82%, 77%, respectively. The pooled sensitivity and specificity of Xpert for TBP compared with CRS were 22% and 99%, respectively. Xpert had a pooled sensitivity of 48% and a pooled specificity of 94% when compared to culture. The results of Xpert were similar to those of previous studies.^[37,38]^ Our results demonstrate that, in an identical population, the sensitivity of Xpert Ultra was better than that of Xpert, but that its specificity was lower. However, significant heterogeneity in sensitivity could still be observed compared to CRS, and heterogeneity exploration revealed that sample type, sample condition, patient selection methods, and homogenization methods might not be a source of heterogeneity. Further analysis was not possible due to data paucity, and large-sample multicenter studies are needed to confirm our observations. A previous comprehensive meta-analysis included some of the TBP data, but that study only reported the results of compared to CRS and not of compared to culture.^[39]^ Another comprehensive meta-analysis that also included some of the TBP data but did not do a head-to-head comparison of Xpert Ultra and Xpert.^[40]^ Our study reported the results of both compared to these 2 references and head-to-head comparison of Xpert Ultra and Xpert for TBP. The range for sensitivity and specificity compared with the 2 references was more plausible, and head-to-head comparison was more credible for comparing the differences between these 2 tests. In addition, our study enrolled more original studies. The CRS varied across studies in present study. Two studies included the results of the index tests in the reference standard, which might also be a source of heterogeneity among the studies.

Although Xpert Ultra had improved the sensitivity for TBP diagnosis, the sensitivity obtained was still imperfect. This might be related to the fact that most of the samples used were of pleural fluid because multiple studies have observed that the sensitivity of Xpert testing was substantially better with pleural tissue than with pleural fluid. As studies that have used Xpert Ultra on pleural tissue are lacking, better performance may be achieved with pleural tissue. Next, while the sensitivity of Xpert Ultra was higher than that of culture, its specificity was lower, which might be related to the paucibacillary nature of pleural fluid. This reduction in specificity can be explained as follows. In samples where culture could not accurately distinguish between infected and non-infected cases, Xpert Ultra detected a large number of infections in culture-negative patients, thus reducing specificity. Xpert Ultra and Xpert were comparable with respect to rifampicin resistance, but as the number of relevant studies and samples were small, further evaluation with larger sample sizes are needed.

This study has a few limitations. This was not a meta-analysis of individual data, and although a systematic search was carried out, it is possible that relevant literature has been missed. Some studies did not provide clear data on TBP, and data from a few studies that compared culture to Xpert systems permitted only descriptive analysis. Even though we attempted to explore sources of heterogeneity, an in-depth analysis was not possible due to paucity of data. Importantly, when CRS was used as the gold standard, significant heterogeneity in sensitivity among studies was evident and, therefore, the results presented need to be treated with caution.

## 5 Conclusions

This study was an independent meta-analysis to head-to-head comparison of the efficacy of Xpert Ultra and Xpert for the diagnosis of TBP. We show that, when compared to CRS or culture, the pooled sensitivity, specificity of Xpert Ultra for TBP diagnosis were 52%, 98%, and 82%, 77%, respectively, and that, similarly, pooled sensitivity and specificity of Xpert, were 22%, 99%, and 48%, 94%, respectively. There was significant heterogeneity in sensitivity among studies compared to CRS. There was insignificant heterogeneity in specificity among studies compared to CRS and insignificant heterogeneity in sensitivity and specificity among studies compared to culture. The sensitivity of the Xpert Ultra was only moderate but better than that of the Xpert, and its specificity was lower than that of the Xpert. The role of Xpert Ultra and Xpert in the early and rapid diagnosis of TBP was limited.

## Acknowledgments

We thank the patients and their families who were included in this study, and our colleagues in the department.

## Author contributions

**Conceptualization:** Da Chen.

**Data curation:** Wenfeng Yu, Yanqin Shen.

**Formal analysis:** Da Chen, Wenfeng Yu.

**Investigation:** Pengfei Zhu, Wenfeng Yu.

**Methodology:** Da Chen, Wenfeng Yu, Yanqin Shen.

**Project administration:** Da Chen.

**Resources:** Pengfei Zhu, Yanqin Shen.

**Software:** Wenfeng Yu, Yanqin Shen.

**Supervision:** Pengfei Zhu.

**Validation:** Yanqin Shen.

**Visualization:** Pengfei Zhu.

**Writing – original draft:** Wenfeng Yu, Yanqin Shen.

**Writing – review & editing:** Da Chen, Wenfeng Yu.

## References

[1] World Health Organization. Global Tuberculosis Report 2020; 2020.

[2] Ronacher K Joosten SA van Crevel R Dockrell HM Walzl G Ottenhoff TH. Acquired immunodeficiencies and tuberculosis: focus on HIV/AIDS and diabetes mellitus. Immunol Rev 2015;264:121–37. 25703556 10.1111/imr.12257

[3] Koenig SP Furin J. Update in tuberculosis/pulmonary infections 2015. Am J Respir Crit Care Med 2016;194:142–6. 27420359 10.1164/rccm.201601-0129UP PMC5003219

[4] Ketata W Rekik WK Ayadi H Kammoun S. Extrapulmonary tuberculosis. Rev Pneumol Clin 2015;71:83–92. 25131362 10.1016/j.pneumo.2014.04.001

[5] Ruan SY Chuang YC Wang JY. Revisiting tuberculous pleurisy: pleural fluid characteristics and diagnostic yield of mycobacterial culture in an endemic area. Thorax 2012;67:822–7. 22436167 10.1136/thoraxjnl-2011-201363 PMC3426072

[6] Shaw JA Irusen EM Diacon AH Koegelenberg CF. Pleural tuberculosis: a concise clinical review. Clin Respir J 2018;12:1779–86. 29660258 10.1111/crj.12900

[7] WHO Guidelines Approved by the Guidelines Review Committee. Xpert MTB/RIF Implementation Manual: Technical and Operational ‘How-To’; Practical Considerations. Geneva: World Health Organization; 2014. 25473699

[8] World Health Organization. Global Tuberculosis Report 2010; 2010.

[9] Denkinger CM Schumacher SG Boehme CC Dendukuri N Pai M Steingart KR. Xpert MTB/RIF assay for the diagnosis of extrapulmonary tuberculosis: a systematic review and meta-analysis. Eur Respir J 2014;44:435–46. 24696113 10.1183/09031936.00007814

[10] Huo ZY Peng L. Is Xpert MTB/RIF appropriate for diagnosing tuberculous pleurisy with pleural fluid samples? A systematic review. BMC Infect Dis 2018;18:284. 29940951 10.1186/s12879-018-3196-4 PMC6019837

[11] Opota O Mazza-Stalder J Greub G Jaton K. The rapid molecular test Xpert MTB/RIF ultra: towards improved tuberculosis diagnosis and rifampicin resistance detection. Clin Microbiol Infect 2019;25:1370–6. 30928564 10.1016/j.cmi.2019.03.021

[12] Chakravorty S Simmons AM Rowneki M. The new Xpert MTB/RIF Ultra: improving detection of *Mycobacterium tuberculosis* and resistance to rifampin in an assay suitable for point-of-care testing. mBio 2017;8:e00812–7. 28851844 10.1128/mBio.00812-17 PMC5574709

[13] Shapiro AE Ross JM Yao M. Xpert MTB/RIF and Xpert Ultra assays for pulmonary tuberculosis and rifampicin resistance in adults irrespective of signs or symptoms of pulmonary tuberculosis. Cochrane Database Syst Rev 2020;3:CD013694. 10.1002/14651858.CD013694.pub2 PMC8437892 33755189

[14] Dorman SE Schumacher SG Alland D. Xpert MTB/RIF Ultra for detection of *Mycobacterium tuberculosis* and rifampicin resistance: a prospective multicentre diagnostic accuracy study. Lancet Infect Dis 2018;18:76–84. 29198911 10.1016/S1473-3099(17)30691-6 PMC6168783

[15] Atherton RR Cresswell FV Ellis J. Detection of *Mycobacterium tuberculosis* in urine by Xpert MTB/RIF Ultra: a useful adjunctive diagnostic tool in HIV-associated tuberculosis. Int J Infect Dis 2018;75:92–4. 30031800 10.1016/j.ijid.2018.07.007 PMC6170999

[16] Bisognin F Lombardi G Lombardo D Re MC Dal Monte P. Improvement of *Mycobacterium tuberculosis* detection by Xpert MTB/RIF Ultra: a head-to-head comparison on Xpert-negative samples. PLoS One 2018;13:e0201934. 30102737 10.1371/journal.pone.0201934 PMC6089413

[17] Meldau R Randall P Pooran A. Same-day tools, including xpert ultra and IRISA-TB, for rapid diagnosis of pleural tuberculosis: a prospective observational study. J Clin Microbiol 2019;57:e00614–9. 31270183 10.1128/JCM.00614-19 PMC6711909

[18] Wang G Wang S Jiang G. Xpert MTB/RIF Ultra improved the diagnosis of paucibacillary tuberculosis: a prospective cohort study. J Infect 2019;78:311–6. 30796951 10.1016/j.jinf.2019.02.010

[19] Moher D Liberati A Tetzlaff J Altman DG. Preferred reporting items for systematic reviews and meta-analyses: the PRISMA statement. PLoS Med 2009;6:e1000097. 19621072 10.1371/journal.pmed.1000097 PMC2707599

[20] 2021;Yu W Shen Y Zhu P Chen D. Head-to-head comparison of the efficacy of Xpert MTB/RIF Ultra and Xpert MTB/RIF for the diagnosis of tuberculous pleurisy: a protocol of systematic review and meta-analysis. 100:e26778.

[21] Whiting PF Rutjes AW Westwood ME. QUADAS-2: a revised tool for the quality assessment of diagnostic accuracy studies. Ann Intern Med 2011;155:529–36. 22007046 10.7326/0003-4819-155-8-201110180-00009

[22] McInnes MDF Moher D Thombs BD. Preferred Reporting Items for a Systematic Review and Meta-analysis of Diagnostic Test Accuracy Studies: the PRISMA-DTA statement. JAMA 2018;319:388–96. 29362800 10.1001/jama.2017.19163

[23] Yu G Zhong F Ye B Xu X Chen D Shen Y. Diagnostic accuracy of the Xpert MTB/RIF assay for lymph node tuberculosis: a systematic review and meta-analysis. Biomed Res Int 2019;2019:4878240. 31236407 10.1155/2019/4878240 PMC6545759

[24] Yu G Shen Y Zhong F Ye B Yang J Chen G. Diagnostic accuracy of the loop-mediated isothermal amplification assay for extrapulmonary tuberculosis: a meta-analysis. PLoS One 2018;13:e0199290. 29944682 10.1371/journal.pone.0199290 PMC6019099

[25] Gao S Wang C Yu X. Xpert MTB/RIF Ultra enhanced tuberculous pleurisy diagnosis for patients with unexplained exudative pleural effusion who underwent a pleural biopsy via thoracoscopy: a prospective cohort study. Int J Infect Dis 2021;106:370–5. 33845198 10.1016/j.ijid.2021.04.011

[26] Wang G Wang S Yang X. Accuracy of Xpert MTB/RIF Ultra for the diagnosis of pleural TB in a multicenter cohort study. Chest 2020;157:268–75. 31437432 10.1016/j.chest.2019.07.027

[27] Wu X Tan G Gao R. Assessment of the Xpert MTB/RIF Ultra assay on rapid diagnosis of extrapulmonary tuberculosis. Int J Infect Dis 2019;81:91–6. 30738907 10.1016/j.ijid.2019.01.050

[28] Wang S. Diagnostic value of xpert MTB /RIF ultra in smear negative pulmonary tuberculosis, tuberculous pleurisy and tuberculous meningitis. Beijing Institute of Tuberculosis and Thoracic Tumor 2019;Chinese.

[29] Melissa M Nicoletta L Antonella L Laura R. Evaluation of Xpert MTB/RIF Ultra assay for rapid diagnosis of pulmonary and extra-pulmonary tuberculosis in an Italian center. Eur J Clin Microbiol Infect Dis 2020;39:1597–600. 32232690 10.1007/s10096-020-03867-y

[30] Sekyere JO Maphalala N Malinga LA Mbelle NM Maningi NE. A comparative evaluation of the new Genexpert MTB/RIF Ultra and other rapid diagnostic assays for detecting tuberculosis in pulmonary and extra pulmonary specimens. Sci Rep 2019;9:16587. 31719625 10.1038/s41598-019-53086-5 PMC6851384

[31] Shaw JA Diacon AH Koegelenberg CFN. Tuberculous pleural effusion. Respirology 2019;24:962–71. 31418985 10.1111/resp.13673

[32] Antonangelo L Faria CS Sales RK. Tuberculous pleural effusion: diagnosis & management. Expert Rev Respir Med 2019;13:747–59. 31246102 10.1080/17476348.2019.1637737

[33] Chitnis AS Davis JL Schecter GF Barry PM Flood JM. Review of nucleic acid amplification tests and clinical prediction rules for diagnosis of tuberculosis in acute care facilities. Infect Control Hosp Epidemiol 2015;36:1215–25. 26166303 10.1017/ice.2015.145

[34] Huggett J Green C Zumla A. Nucleic acid detection and quantification in the developing world. Biochem Soc Trans 2009;37:419–23. 19290873 10.1042/BST0370419

[35] Yu G Ye B Chen D. Comparison between the diagnostic validities of Xpert MTB/RIF and interferon-γ release assays for tuberculous pericarditis using pericardial tissue. PLoS One 2017;12:e0188704. 29211755 10.1371/journal.pone.0188704 PMC5718425

[36] Piersimoni C Gherardi G Gracciotti N Pocognoli A. Comparative evaluation of Xpert MTB/RIF and the new Xpert MTB/RIF ultra with respiratory and extra-pulmonary specimens for tuberculosis case detection in a low incidence setting. J Clin Tuberc Other Mycobact Dis 2019;15:100094. 31720421 10.1016/j.jctube.2019.100094 PMC6830143

[37] Kohli M Schiller I Dendukuri N. Xpert^®^ MTB/RIF assay for extrapulmonary tuberculosis and rifampicin resistance. Cochrane Database Syst Rev 2018;8:CD012768. 30148542 10.1002/14651858.CD012768.pub2 PMC6513199

[38] Sehgal IS Dhooria S Aggarwal AN Behera D Agarwal R Land GA. Diagnostic performance of Xpert MTB/RIF in tuberculous pleural effusion: systematic review and meta-analysis. J ClinJ Microbiol 2016;54:1133–6. 10.1128/JCM.03205-15 PMC4809962 26818675

[39] Jiang J Yang J Shi Y. Head-to-head comparison of the diagnostic accuracy of Xpert MTB/RIF and Xpert MTB/RIF Ultra for tuberculosis: a meta-analysis. Infect Dis (Lond) 2020;52:763–75. 32619114 10.1080/23744235.2020.1788222

[40] Kohli M Schiller I Dendukuri N. Xpert MTB/RIF Ultra and Xpert MTB/RIF assays for extrapulmonary tuberculosis and rifampicin resistance in adults. Cochrane Database Syst Rev 2021;15:CD012768. 10.1002/14651858.CD012768.pub3 PMC8078545 33448348

